# Barriers and facilitators to Parkinson’s disease research participation amongst underrepresented groups

**DOI:** 10.1186/s13104-025-07293-1

**Published:** 2025-05-29

**Authors:** Jennifer Adrissi, Anabel Marre, Maxwell Edwin Shramuk, Emily Zivin, Karen Williams, Danielle Larson

**Affiliations:** 1 Department of Neurology, University of California Los Angeles, 1100 Glendon Avenue 11th Floor, Los Angeles, CA 90024 USA; 2 Northwestern University, 633 Clark Street, Evanston, IL 60208 USA; 3 Department of Preventive Medicine, Northwestern University Feinberg School of Medicine, 680 N Lake Shore Drive Suite 1400, Chicago, IL 60611 USA; 4 Department of Neurology, Northwestern University Feinberg School of Medicine, 710 Lake Shore Drive 11th Floor, Chicago, IL 60611 USA

**Keywords:** Underrepresented groups, Parkinson’s disease, Clinical research, Community-academic partnership

## Abstract

**Objective:**

Even though the growing prevalence of Parkinson’s disease (PD) is inclusive of ethnic and racial minority groups, these populations remain underrepresented in PD clinical research. This community-based study seeks to add to the limited knowledge on barriers and facilitators to underrepresented group (URG) enrollment in PD trials by assessing minority community members’ PD and research knowledge, trust in medical researchers, and likelihood to participate in research based on various study design factors.

**Results:**

Of the 97 total workshop participants, 80 completed demographic information, with the majority female (71%) and from minority racial groups -- African American/Black (37.5%) and East/Southeast Asian (45%). Levels of trust in medical researchers were generally high and improved post-workshop. Most respondents were likely to participate in trials requiring DNA or cognitive testing, and unlikely if requiring intravenous infusion or lumbar puncture. Facilitators to trial participation included offering transportation and financial incentives, while longer study visits and study duration were barriers.

**Supplementary Information:**

The online version contains supplementary material available at 10.1186/s13104-025-07293-1.

## Introduction

As of 2020, Parkinson’s disease (PD) is the second most common neurodegenerative disease in the U.S. The yearly incidence of PD is rising, with nearly 90,000 people aged 65 or older diagnosed in the country each year [1]. PD affects all races and ethnicities, with individuals identifying with one or more minority group accounting for at least one-fifth of people with PD in the U.S [2]. With the increasing number of individuals living with PD comes strain on the healthcare system, underscoring the urgent need for more effective treatments and disease-modifying therapies. Clinical research is vital to achieve this goal; however, the historically homogenous participant makeup of PD clinical trials threatens to produce results that are not generalizable to the diverse PD population [3, 4]. Less than 10% of participants in U.S. PD clinical trials are from racial and ethnic minority groups [5]. All racial and ethnic groups aside from non-Hispanic White people are historically underrepresented in PD research, with past research highlighting this disparity in Black, Hispanic/Latino, and Asian communities [5, 6].

Investigating why this disparity exists is key to improving underrepresented group (URG) enrollment in PD clinical trials. There are several hypotheses which include a combination of incommensurate healthcare access and delayed PD diagnosis [7–9], lower health literacy and awareness of research [10, 11], and mistrust of medical research [12, 13] amongst minority populations. This community-based quasi-interventional study adds to the limited understanding of contributors to inequitable PD research enrollment by investigating knowledge and perceptions of PD and PD research, as well as identifying barriers and facilitators to research participation in predominantly minority communities of Chicago.

## Methods

### Study design and recruitment

This was a quasi-interventional study evaluating the perceptions of URGs toward PD and PD clinical research engagement and the effects of an educational intervention on these perceptions. This study was developed through the Chicago Movement Coalition (CMC), a community-academic partnership based on a community-based participatory research (CBPR) framework [14]. The CMC is led by an advisory council of Movement Disorders neurologists, community leaders, people with Parkinson’s (PWPs), and care partners from historically understudied groups. Members of the CMC advisory council proposed eight community partner organizations who were comprised of and/or worked with URGs in Chicago. The CMC then partnered with these organizations to host educational workshops, sharing information about PD and PD clinical research.

In collaboration with the leadership of the community partners, community-dwelling individuals aged 18 years and older, with or without Parkinson’s disease, were recruited and invited to the educational workshop. Given the community-based framework and exploratory nature of the study, convenience sampling was used. Workshop participants completed a pre-workshop survey assessing baseline knowledge and perceptions of PD and clinical research. After the educational presentation, which included information about PD prevalence, symptoms, management options and basics of clinical research, participants were given a similar post-workshop survey to assess the impact of the educational intervention on participant perspectives. Three of the eight workshops took place in-person at the community organization space. The remaining were held virtually via video conferencing (Zoom). Participants received up to $20 in compensation upon completion of the research surveys. Workshops were held between February and November 2021. The study was approved by the Institutional Review Board of Northwestern University. Survey data were collected and managed with REDCap (Research Electronic Data Capture), a secure, web-based software platform designed to support data capture for research studies, hosted at Northwestern University [15].

### Data collection and analysis

The Pre-Workshop Survey included (Appendix A):

- Demographic information.
- PD Knowledge Assessment: 5 multiple choice questions, 1 free response.
- Trust in Medical Researchers Scale (TIMRS).
- Penn PD Research Participation (PPRP) Survey.

The Post-Workshop Survey included (Appendix B):

- PD Knowledge Assessment (identical to Pre-Workshop Assessment).
- Three multiple-choice workshop feedback questions.
- TIMRS
- PPRP Survey.

The TIMRS is a 12-item Likert-based survey which has been validated to measure trust in medical research recruitment and engagement [16]. The composite scale yields scores from 0 to 48, with higher scores indicating a greater trust in medical researchers.

The PPRP survey presents three hypothetical PD clinical trial protocols and asks 18 questions with Likert-based response options to gauge interest in different aspects and types of clinical research [17].

## Results

### Demographics

Demographics of respondents are shown in Table 1. The most represented races were African American or Black (37.5%) and East or Southeast Asian (45%). This was a study of the general public, not specifically those with PD. Of those surveyed, only one participant had a PD diagnosis; however, 10.8% had a family member with PD and almost half (44.2%) knew someone personally who had PD.

**Table 1:** Participant demographics

Characteristic	Both Surveys, *N* = 19	Post-Survey Only, *N* = 3	Pre-Survey Only, *N* = 58	Overall, *N* = 80
**Gender Identity**; n (%)				
Female	13 (68.42%)	2 (66.67%)	42 (72.41%)	57 (71.25%)
Male	6 (31.58%)	1 (33.33%)	16 (27.59%)	23 (28.75%)
**Self-identified Race;** n (%)				
African American or Black	8 (42.11%)	0 (0.00%)	22 (37.93%)	30 (37.50%)
Asian - East or Southeast	6 (31.58%)	2 (66.67%)	28 (48.28%)	36 (45.00%)
Asian - South	0 (0.00%)	0 (0.00%)	1 (1.72%)	1 (1.25%)
White	5 (26.32%)	1 (33.33%)	4 (6.90%)	10 (12.50%)
Multi-racial or Unknown	0 (0.00%)	0 (0.00%)	3 (5.17%)	3 (2.75%)
**Self-identified Ethnicity**; n (%)				
Hispanic or Latino	1 (5.26%)	1 (33.33%)	0 (0.00%)	2 (2.50%)
Not Hispanic or Latino	15 L (78.95%)	2 (66.67%)	54 (93.10%)	71 (88.75%)
Decline to answer	2 (10.53%)	0 (0.00%)	1 (1.72%)	3 (3.75%)
Unknown	1 (5.26%)	0 (0.00%)	3 (5.17%)	4 (5.00%)
**Highest level of Education**; n (%)				
12th grade or less	0 (0.00%)	0 (0.00%)	4 (6.98%)	4 (5%)
HS graduate/ GED	1 (5.26%)	0 (0.00%)	9 (15.52%)	10 (12.50%)
Post-HS vocational, technical or trade school, some college	4 (21.05%)	1 (33.33%)	15 (25.86%)	20 (25.0%)
Associates or Bachelor’s degree	9 (47.37%)	1 (33.33%)	15 (25.867%)	25 (31.25%)
Masters, Professional or Doctoral degree	5 (26.31%)	1 (33.33%)	15 (25.87%)	21 (26.25%)
**Total Household Income;** n (%)				
Less than $35,000	3 (15.79%)	0 (0.00%)	18 (31.03%)	21 (26.25%)
$35,000 to $74,999	8 (42.11%)	1 (33.33%)	19 (32.76%)	28 (35%)
$75,000 to $99,999	2 (10.53%)	0 (0.00%)	5 (8.62%)	7 (8.75%)
More than $100,000	2 (10.53%)	1 (33.33%)	7 (12.07%)	10 (12.50%)
Prefer not to answer	4 (21.05%)	1 (33.33%)	9 (15.52%)	14 (17.50%)

### PD and clinical trial knowledge assessment

69% of respondents were able to identify tremor as a common early symptom of PD, but 10.9% incorrectly stated numbness, and 14.1% incorrectly stated memory loss were common early symptoms. Most survey respondents (95.6%) were able to identify some of the treatment categories for PD (medications, physical therapy, exercise). Only about half knew that there is no current cure for PD and that there is no blood test to diagnose PD, 55.9% and 52.9%, respectively. There was no significant improvement in the percentage of correct responses from pre-workshop to post-workshop assessments.

When asked about prior participation in clinical research trials, 26% of respondents marked that they had participated in a clinical research trial, and 5% of those were trials for PD or PD-related symptoms. Black respondents had higher rates of participation in clinical research trials in any discipline than Asian respondents, 34.4% vs. 14.7%. Before this study, only 8.1% of respondents had ever been asked by a researcher or medical provider to participate in a PD-specific research trial, as a participant with PD or control participant.

### TIMRS

**Fig. 1:**
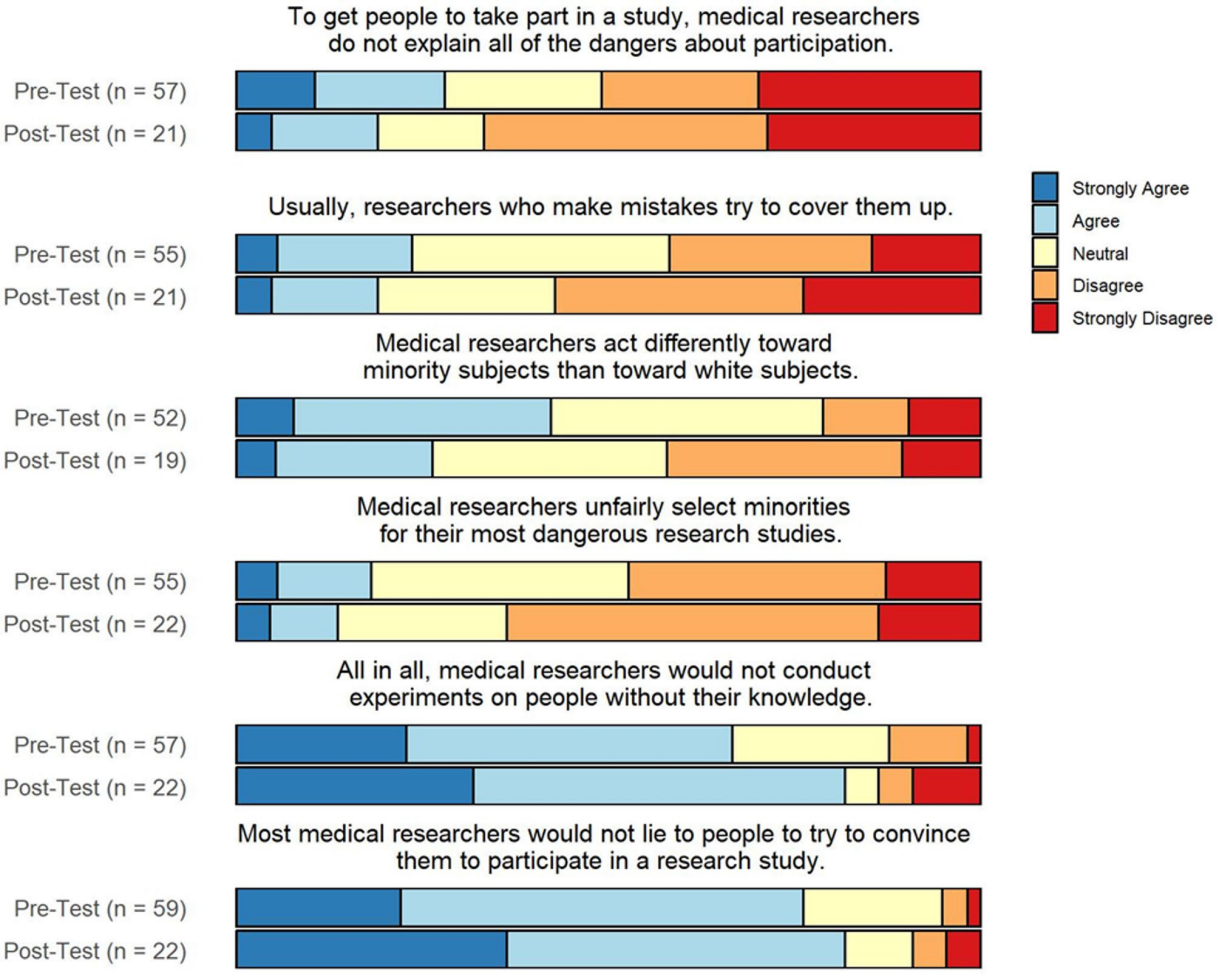
Pre- and post-workshop TIMRS responses* *Pre-workshop responses were pooled to include participants that completed both pre- and post-workshop surveys or only the pre-workshop survey. Likewise, post-workshop responses were pooled to include participants that completed both pre- and post-workshop surveys or only the post-workshop survey

Combined responses to six of the 12 TIMRS questions are shown in Fig. 1. The five response options ranged from strongly disagree to strongly agree (scores 0–4), with the response associated with the least trust assigned 0 and the most trust assigned 4. Since the post-workshop scores are higher than 24, which is the score given if the responses to all questions are neutral, this is consistent with a higher level of trust (Appendix C).

### PPRP

**Fig. 2:**
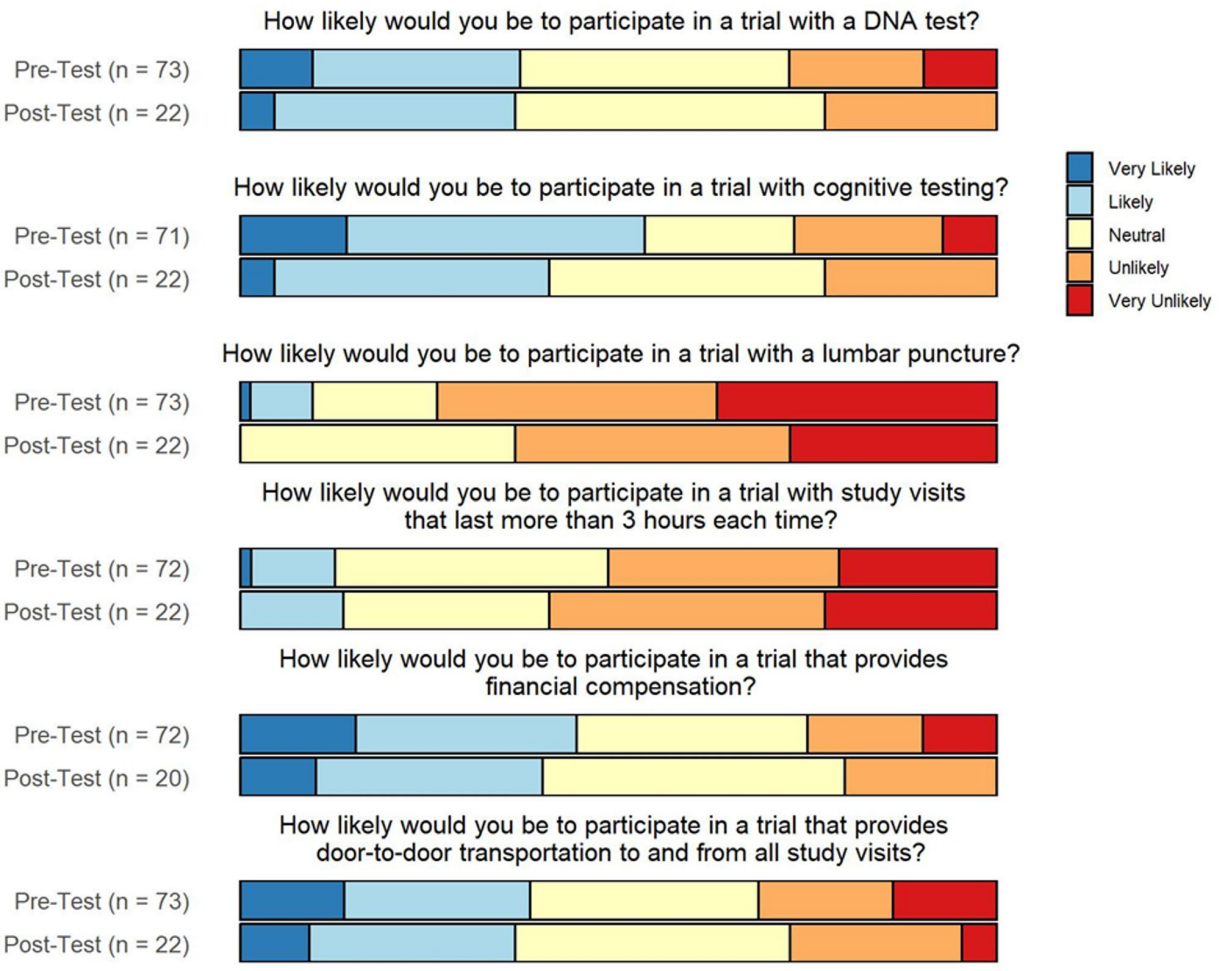
Pre- and post-workshop PPRP responses* *Pre-workshop responses were pooled to include participants that completed both pre- and post-workshop surveys or only the pre-workshop survey. Likewise, post-workshop responses were pooled to include participants that completed both pre- and post-workshop surveys or only the post-workshop survey

Combined responses to six of the 18 PPRP questions are shown in Fig. 2. On pre-assessment, 70% of respondents were unlikely or very unlikely to participate if the study involved an intravenous infusion. Comparatively, there was slightly more willingness to participate if it involved a daily pill, with 54.2% being unlikely or very unlikely. Most respondents were very likely, likely, or neutral to participating in trials requiring a DNA test (72.61% pre- and 76.19% post-workshop) or cognitive testing (73.24% pre- and 76.19% post-workshop).

With respect to study logistics, few participants (12.5%) were likely or very likely to participate in a study with visits more than 3 h each, increasing slightly to 14.3% on post-assessment. There was also a low percentage of participants likely or very likely to participate in in-person study visits lasting more than a year, about 19% on both pre- and post-assessments.

### Workshop feedback

Thirty-eight of the workshop participants answered workshop feedback questions. Thirty-six of the 38 participants (95%) felt more comfortable in their understanding of PD signs and symptoms after the workshop. Thirty-four (89%) reported increased knowledge of PD resources after the workshop. All but one participant were satisfied with workshop structure and content.

## Discussion

The results of this quasi-interventional study add to the limited existing literature on perceptions, facilitators, and barriers to PD research participation among minority populations, specifically Black and Asian communities. Possible barriers identified include lack of knowledge of PD signs and symptoms and lack of awareness of clinical research opportunities. Previous literature has shown that individuals from URGs have lower levels of trust in medical research; however, lack of trust in medical researchers was *not* identified as a major barrier in this study. Survey results revealed that facilitators to research participation included a convenient, patient-centered, minimally invasive study design with appropriate participant compensation.

### Lack of PD knowledge

The findings from this study echo previous reports that suggest individuals from minority groups may have less awareness of PD symptoms and diagnosis and are more likely to attribute PD symptoms to normal signs of aging rather than signs of a neurological condition [18, 19]. Less than three-quarters of the respondents in our study correctly identified early PD symptoms and approximately half correctly indicated that there is no current blood test or cure for PD. This highlights the need for community education to raise awareness of PD signs, symptoms, and diagnosis to ensure that the underdiagnosis of PD in minority populations is not a significant barrier to their care and potential research participation.

### Lack of awareness of PD research

Our survey results revealed that 26% of respondents had previously participated in a clinical research trial, and only 8.1% of respondents had been asked by a researcher or medical provider to participate in a PD-specific research trial. Limited awareness of research has been previously cited as a barrier to research participation among Black Americans [20] with similar findings also reported in Hispanic populations. Despite being interested in research, Hispanic PWP and care partners not linked to a tertiary medical center were largely unaware of research opportunities [11, 21]. This highlights underlying contributors to insufficient research awareness– lack of engagement of community health providers and inadequate access to movement disorder specialists and centers where PD research opportunities are promoted and conducted.

Black Americans are half as likely to be diagnosed with PD as White Americans, even after controlling for age, sex, and location of care and healthcare use [22, 23]. Additionally, women and non-White individuals that are diagnosed are less likely to be treated by a neurologist [9]. Therefore, increasing referrals to specialized PD centers and decreasing access barriers are vital steps toward improving URG research participation. Furthermore, information about PD and PD research must extend beyond the walls of tertiary care centers and reach the community itself.

### Impact of trust on research participation

Importantly, our survey results demonstrate a moderate level of trust in medical research amongst the sample, including Black and Asian subgroups, with mean post-workshop composite and sub-scale TIMRS scores higher than 24, associated with higher levels of trust and greater likelihood to participate in research [16]. This challenges a common argument that minority groups do not participate in research because they are distrustful. Similarly contrasting this assumption, a recent study of Hispanic perspectives on PD research participation demonstrated high levels of trust amongst the Hispanic population [11]. While decreased trust caused by historical and current injustices in medicine and research likely affect the propensity toward research engagement, our findings suggest that the magnitude of this contribution may be overemphasized, distracting from other addressable barriers and factors affecting under-enrollment of URGs in PD trials.

### Impact of study design

Survey results highlighted important study design factors for URGs that substantiate trends in previous literature. Firstly, convenience factors are likely to increase enrollment, such as providing transportation or conducting visits in-home, conducting shorter study visits, and not requiring a care partner at every visit. This echoes similar findings in a study that showed that Black people rated the requirement of a study partner and study location as more important factors in the decision to enroll than did White respondents [24]. Compensation was identified as an incentive for research participation, as has been previously reported [13]. The degree of invasiveness of the study procedures influences individuals’ willingness to participate, as the majority were unlikely to participate if a lumbar puncture (LP) was required or if the study involved an intravenous infusion medication. Individuals were largely willing to participate in trials involving DNA testing, a relevant finding given the growing amount of genetic-based PD trials.

### Limitations

There were several limitations to this small, quasi-interventional study. Firstly, there was drop-off of participants from pre- to post-workshop surveys (less than half of the participants completed the post-workshop evaluation), which limited sample size and opportunities for paired analysis. A community-partnered approach resulted in recruitment of the targeted population, but the convenience sampling could contribute to sample bias, although the effect is likely minimal. The predominantly Black and Asian study population limits the generalization of findings to all URGs in PD research. Furthermore, generalization of findings to individuals with lower levels of education is limited, given the study sample’s high rate of post-secondary degrees (57.5%, Table 1). Most participants in this study did not have PD, so while the information gathered can inform our understanding of perceptions and preferences of potential control participants in PD trials, future studies specifically investigating the perceptions of PWP from URGs are needed.

## Conclusion

This study highlights key barriers and facilitators to PD research participation among predominately Black and Asian communities, including limited knowledge of PD symptoms and low awareness of PD research opportunities. While historical mistrust in medical research is often cited as a barrier, our findings suggest that it may be overemphasized compared to other addressable factors such as duration of study visits, transportation costs, appropriate compensation, and community-centered recruitment efforts. One potential approach to future interventions includes using CBPR approaches to foster academic-community partnerships and infrastructure in underserved communities [25, 26]. Larger-scale, multi-center studies are necessary to better elucidate the multiple contributions to the underrepresentation of minority groups in medical research and develop interventions to increase minority enrollment in PD clinical trials.

## Electronic supplementary material

Below is the link to the electronic supplementary material.

Supplementary Material 1

Supplementary Material 2

Supplementary Material 3

## Data Availability

The data used during the current study are available from the corresponding author on reasonable request.

## Abbreviations

CBPR: Community-Based Participatory Research
CMC: Chicago Movement Coalition
PD: Parkinson’s disease
PPRP: Penn PD Research Participation Survey
PWP: People with Parkinson’s
TIMRS: Trust in Medical Researcher Scale
URG: Underrepresented group
