# Supplementary material for: Barriers and facilitators to Parkinson’s disease research participation amongst underrepresented groups
Source: BMC Res Notes. 2025 May 29;18:240. doi: 10.1186/s13104-025-07293-1 (PMC12121102; doi:10.1186/s13104-025-07293-1)
Supplement: Supplementary file 2 — Supplementary Material 2 [file 13104_2025_7293_MOESM2_ESM.docx]

|  | **TIMRS Composite Score** | | **Participant Deception Sub-Score** | | **Research Honesty Sub-Score** | |
| --- | --- | --- | --- | --- | --- | --- |
| **Race** | **Pre** | **Post** | **Pre** | **Post** | **Pre** | **Post** |
| **All** | **27.42** SD = 7.49  n = 40 | **31.56** SD = 8.1  n = 18 | **24.7** SD = 10.34  n = 43 | **28.44** SD = 10.92  n = 18 | **31.29** SD = 7.07  n = 51 | **33.82** SD = 7.83  n = 22 |
| **Black** | **23.11** SD = 7.1  n = 9 | **30** SD = 7.27  n = 6 | **19.67** SD = 9.26  n = 12 | **24.67** SD = 9.93,  n = 6 | **30.55** SD = 7.54  n = 11 | **31.5** SD = 9.67  n = 8 |
| **White** | **38** SD = 7.31  n = 5 | **38.75** SD = 7.18  n = 4 | **40.4** SD = 4.98  n = 5 | **40** SD = 7.3,  n = 4 | **37.5** SD = 9.18  n = 8 | **38.33** SD = 5.72  n = 6 |
| **Asian** | **27.08** SD = 5.82  n = 25 | **29.12** SD = 7.83  n = 8 | **23.92** SD = 8.88  n = 25 | **25.5** SD = 9.9,  n = 8 | **30.34** SD = 5.43  n = 29 | **32.75** SD = 6.5  n = 8 |

***Table 2:*** *Pre- and Post-Workshop TIMRS Scores*

Mean scores for the composite TIMRS and Participant Deception and Research Honesty sub-scales are shown. Maximum score for the composite and sub-scales is 48.
