# Supplementary material for: Barriers and facilitators to Parkinson’s disease research participation amongst underrepresented groups
Source: BMC Res Notes. 2025 May 29;18:240. doi: 10.1186/s13104-025-07293-1 (PMC12121102; doi:10.1186/s13104-025-07293-1)
Supplement: Supplementary file 3 — Supplementary Material 3 [file 13104_2025_7293_MOESM3_ESM.docx]

**Pre-Workshop Assessment**

Please choose:

**Gender Identity:** Male / Female / Non-Binary / Other

**Age:** Under 30 / 30 – 50 / 51 – 70 / Over 70

**Racial Identity:** Caucasian / American Indian, Alaska Native / African American, Black, West Indian, Afro-Caribbean, Afro-Latino, African / Asian – East or Southeast / Asian - South / Native Hawaiian or Pacific Islander / Other: _____________

**Ethnic Identity:** Hispanic or Latino / Not Hispanic or Latino

**Highest Education Level:** Less than High School or High School – Did not graduate / High School Graduate / Some post-High School Training / Associate Degree / Bachelor’s Degree / Graduate Degree

**Total Household Income:** Less than $25,000 / $25,001 - $50,000 / $50,001 - $75,000 / $75,001 - $100,000 / More than $100,000

Please select the correct answer to the following questions.

1. Parkinson’s disease is contagious: **True** or **False**
2. There is a blood test to diagnose Parkinson’s disease: **True** or **False**
3. There is no cure for Parkinson’s disease: **True** or **False**
4. Which of the following is a common early symptom of Parkinson’s disease?
   1. **Weakness on one side of the body**
   2. **Tremor**
   3. **Numbness**
   4. **Memory loss**
5. Which of the following is helpful with treating Parkinson’s disease symptoms?
   1. **Medications**
   2. **Physical therapy**
   3. **Exercise**
   4. **All of the above**

**What do you think of when you hear *Parkinson’s disease*?**

**How would you describe Parkinson’s disease and what it looks like?**

**Penn PD Research Participation (PPRP) Survey**

| **CURRENT MARITAL STATUS. Are you currently married, living with a partner, separated, divorced, widowed, or never married?** | | |
| --- | --- | --- |
| - MARRIED |  | since what year? _____________ |
| - LIVING WITH A PARTNER |  | since what year? _____________ |
| - SEPARATED / DIVORCED |  | since what year? _____________ |
| - WIDOWED |  | since what year? _____________ |
| - NEVER MARRIED |  |  |

| **ARE YOU A CAREGIVER OR CAREPARTNER FOR ANYONE with Parkinson’s disease**?  By caregiver/care partner we mean YOU help someone else on most days with activities such as dressing, bathing, eating, cooking, transportation, medications, shopping or are a companion/ sitter. This person could be a spouse, child, grandchild, parent, etc. | - Yes - No |
| --- | --- |

| **HOW MANY PEOPLE LIVE in YOUR HOUSEHOLD besides you?__________** | |
| --- | --- |
| **How many are children (ages 0-18) ____________** | |
| **WHO LIVES WITH YOU? Check all** | |
| - SPOUSE/PARTNER | - AUNT (S) /UNCLE (S) |
| - CHILD/CHILDREN /STEPCHILD | - NEICE(S)/NEPHEW(S) |
| - PARENTS | - PAID AIDE/ HOUSEKEEPER |
| - GRANDCHILD/ GRANDCHILDREN | - FRIEND(S) OR ROOMMATE(S) |
| - SIBLING(S) | - SOMEONE ELSE |

| **HOW FAR DO you live from your closest Parkinson disease research center?** |
| --- |
| - Less than 5 miles |
| - 6-10 miles |
| - 11-15 miles |
| - 16-25 miles |
| - More than 25 miles |
| - I don’t know |

| **What is your gender identity?** |
| --- |
| - Female |
| - Male |
| - Non-binary |
| - Other |

| **What is your total household income?** |
| --- |
| - Less than $20,000 |
| - $20,000 to $34,999 |
| - $35,000 to $49,999 |
| - $50,000 to $74,999 |
| - $75,000 to $99,999 |
| - More than $100,000 - Prefer not to answer |

| **ETHNICITY**: Which category of ethnicity do you most closely identify with?  *Ethnicity is a category of people who identified with each other, usually on the basis on similarities such as common languages, history, society, culture or nation.*  *NOTE: Race is a different category, you will also have an opportunity to self-identify in this category.* | | | |
| --- | --- | --- | --- |
| - Hispanic or Latino | - Not Hispanic or Latino | | |
| If you **are** Hispanic or Latino, specify origin: | | | |
|  |  | | |
| **RACE**: Please place an "X" next to **all** racial categories you identify with. If you are able to specify with more detail, please enter information in the box below your selected categories.  *Race is associated with biological factors and is separate from ethnicity. It refers to a person’s physical characteristics, such as bone structure and skin, hair, or eye color.* | | | |
| - American Indian/Alaska Native   *Specify origin(s)—for example, Navajo Nation, Blackfeet tribe, Muscogee (Creek) Nation, Mayan, etc.*   \|  \| \| --- \| | | | |
| - African American, Black, West Indian, Afro-Caribbean, Afro-Latino, African (sub-Saharan)   *Specify origin(s)—for example,* *African American, Jamaican, Haitian, Cuban, Colombian, Brazilian, Nigerian, Ethiopian, etc.*   \|  \| \| --- \| | | | |
| - Asian - East or Southeast   *Specify origin(s)—for example, China, Japan, North or South Korea, Vietnam, Thailand, Philippines, Cambodia, Singapore etc.*   \|  \| \| --- \| | | | |
| - Asian - West or Southwest   *Specify origin(s)—for example, the Middle East, Bangladesh, Iran, Iraq, Syria, Saudi Arabia, Qatar, United Arab Emirates, Yemen, etc.*   \|  \| \| --- \| | | | |
| - Asian - South   *Specify origin(s)—for example, Bangladesh, India, Pakistan, Sri Lanka, etc.*   \|  \| \| --- \| | | | |
| - Caucasian, White   *Specify origin(s)—for example, British Isles, Germany, Spain, France, Italy, Sweden, etc.*   \|  \| \| --- \| | | | |
| - Native Hawaiian, Other Pacific Islander   *Specify origin(s)—for example, Native Hawaiian, Samoan, Guamanian or Chamorro, Tongan, etc.*   \|  \| \| --- \| | | | |
| - Some other race or origin  \|  \| \| --- \| | | | |
| **EDUCATION LEVEL (select the highest level attained):** | | | |
| - Never attended/ Kindergarten only | | | |
| - 1st-8th grade | | | |
| - 9th-12th grade (no diploma) | | | |
| - High school graduate/ GED | | | |
| - Post High School vocational, technical or trade school | | | |
| - Some college but no degree | | | |
| - Associate’s degree | | | |
| - Bachelor’s degree | | | |
| - Master’s degree | | | |
| - Professional or doctoral degree | | | |
| **PARKINSON’S DIAGNOSIS AND SYMPTOM ONSET.** | | |  |
| In what year did a medical provider first tell you that you had or possible had Parkinson's disease | | **YEAR**  **DIAGNOSED**=____________ |  |
| In what year did you start having symptoms that you think were due to Parkinson’s? | | **YEAR**  **SYMPTOM**  **ONSET**= _______________ |  |

| **FAMILY HISTORY**: Have any of the following relatives/ family members been diagnosed with Parkinson Disease, Parkinson’s or parkinsonism?  CHECK ALL THAT APPLY | |
| --- | --- |
| - Mother | - Maternal Grandparent (s) |
| - Father | - Paternal Grandparent (s) |
| - Brother (s) | - Aunt (s) or Uncles (s) |
| - Sister (s) | - Cousin (s) |
| - None of these relatives have been diagnosed with this condition. - I don’t know | |

| **Are you currently working or the primary caregiver for someone?** |
| --- |
| - YES, FULL TIME (>30 hours per week) |
| - YES, PART TIME (less than 30 hours per week) |
| - NO |

| **IF you are working, do you have flexibility to leave work for person reasons or to rearrange your work hours? For example, if you were participating in a research study, could you miss work for study visits or rearrange your schedule without losing pay or otherwise being penalized (having to take sick days or vacation days)?** |
| --- |
| - My job would allow me to leave work for research study visits without penalty |
| - My job would allow me to leave work for research study visits without, but I may need to take sick days or vacation days. |
| - My job would not allow me to rearrange my work schedule to accommodate medical research visits. |

| **Have you ever participated in a clinical research trial?** | |
| --- | --- |
| - YES | - NO |
| **Was the trial for PD or a PD related symptom?** | |
| - YES | - NO |
| **Before this study, has a researcher or medical provider ever asked you to participate in a PD research trial?** | |
| - YES | - NO |

These questions refer to the following hypothetical/pretend research trial.

**TRIAL SCENARIO A**

A national research trial will test whether a new medication for Parkinson disease is safe and effective. The previous studies of this medication have been done in the lab. Scientists do not know if the drug is safe or works in humans. If it works, it will slow down the progression of Parkinson disease.

TYPE OF STUDY: TREATMENT

POTENTIAL RISKS: WE DO NOT KNOW IF THE DRUG IS SAFE OR WORKS IN HUMANS. IT HAS BEEN TESTED IN THE LAB, AND LOOKS SAFE AND EFFECTIVE IN OTHER ANIMALS

POTENTIAL BENEFITS: IT MAY SLOW DOWN OR STOP PARKINSON DISEASE. IT WILL NOT REVERSE IT.

| Based on the information above, how likely would you be to participate in the trial? Answer based on your current level of interest, symptoms and functional ability. | - Extremely unlikely to enroll |
| --- | --- |
|  | - Unlikely to enroll |
|  | - Neutral |
|  | - Likely to enroll |
|  | - Extremely likely to enroll |

These questions refer to the following hypothetical/pretend research study.

**TRIAL SCENARIO B**

A national research study will follow Parkinson disease progression to understand how PD changes over time and is different for different people. There are no extra treatments or study drugs involved. Research data is collected over time.

TYPE OF STUDY: OBSERVATION

POTENTIAL RISKS: YOU MAY GET TIRED OF OR ANNOYED BY STUDY TESTS (SURVEYS, BLOOD DRAWS, IMAGING, ETC.)

POTENTIAL BENEFITS: WE WILL LEARN MORE ABOUT YOUR CONDITION

| Based on the information above, how likely would you be to participate in the trial? Answer based on your current level of interest, symptoms and functional ability. | - Extremely unlikely to enroll |
| --- | --- |
|  | - Unlikely to enroll |
|  | - Neutral |
|  | - Likely to enroll |
|  | - Extremely likely to enroll |

These questions refer to the following hypothetical/pretend research study.

**TRIAL SCENARIO C**

A national research study will follow people who are at risk for Parkinson disease. Researchers want to find a way to detect Parkinson disease earlier, maybe even before there are symptoms. There are no extra treatments or study drugs involved. Research data is collected over time.

TYPE OF STUDY: OBSERVATIONAL

POTENTIAL RISKS: YOU MAY GET TIRED OF OR ANNOYED BY STUDY TESTS (SURVEYS, BLOOD DRAWS, IMAGING, ETC.). YOU MAY FIND OUT YOU ARE VERY LIKELY TO HAVE PARKINSON DISEASE IN THE FUTURE, EVEN THOUGH YOU FEEL FINE NOW.

POTENTIAL BENEFITS: WE MAY LEARN HOW TO DETECT PD, BEFORE IT CAUSES SYMPTOMS. WE MAY FIND NEW POTENTIAL WAYS TO TRY TO STOP PD BEFORE IT CAUSES SYMPTOMS.

| Based on the information above, how likely would you be to participate in the trial? Answer based on your current level of interest, symptoms and functional ability. | - Extremely unlikely to enroll |
| --- | --- |
|  | - Unlikely to enroll |
|  | - Neutral |
|  | - Likely to enroll |
|  | - Extremely likely to enroll |

Please rate whether each of the following study design factors would make you more or less likely to participate in this trial (CHECK ONE ANSWER FOR EACH OPTION)

| The trial requires participants to undergo **DNA or genetic testing**. This involves swabbing the mouth with a cotton swab. The results will be stored in a research database, so we can learn more about genes that might cause PD. The genetic information will be kept anonymous. | - Much less likely to enroll |
| --- | --- |
|  | - Less likely to enroll |
|  | - Neutral |
|  | - More likely to enroll |
|  | - Much more likely to enroll |
| The trial requires participants to undergo **blood testing/provide blood specimens, mostly to make sure you are not having an adverse reaction to the study drug**. This involves piercing the skin with a needle and collecting blood in a small tube. | - Much less likely to enroll |
|  | - Less likely to enroll |
|  | - Neutral |
|  | - More likely to enroll |
|  | - Much more likely to enroll |
| The trial requires participants to undergo **cognitive testing**. This involves answering questions, working puzzles, and doing mental tasks for at least one hour. The test is designed to identify problems with thinking or memory. | - Much less likely to enroll |
|  | - Less likely to enroll |
|  | - Neutral |
|  | - More likely to enroll |
|  | - Much more likely to enroll |
| The trial requires participants to undergo **MRI** **imaging.** This involves receiving imaging contrast in your vein, and then lying still and flat on your back in an MRI scanner for up to an hour while pictures of your brain are taken. An MRI scanner leaves about one foot of space above your face. The MRI may show brain disease other than Parkinson disease. | - Much less likely to enroll |
|  | - Less likely to enroll |
|  | - Neutral |
|  | - More likely to enroll |
|  | - Much more likely to enroll |
| The trial requires participants to undergo a **DAT scan.** This imaging test involves receiving a small amount of nuclear radiation in the arm vein, then lying still on your back for about an hour. Some researchers think the DAT scan can determine if you have Parkinson disease, or may develop it in the future. | - Much less likely to enroll |
|  | - Less likely to enroll |
|  | - Neutral |
|  | - More likely to enroll |
|  | - Much more likely to enroll |

| The trial requires participants to undergo at least one **lumbar puncture.** During a lumbar puncture, a needle is inserted between two bones lower back/spine, and a sample of cerebrospinal fluid is removed. The procedure takes 45 minutes, and you may have back pain, headache afterwards. | | | - Much less likely to enroll | |
| --- | --- | --- | --- | --- |
|  |  |  | - Less likely to enroll | |
|  |  |  | - Neutral | |
|  |  |  | - More likely to enroll | |
|  |  |  | - Much more likely to enroll | |
| The trial has study visits that last more than 3 hours each time. | | - Much less likely to enroll | | |
|  |  | - Less likely to enroll | | |
|  |  | - Neutral | | |
|  |  | - More likely to enroll | | |
|  |  | - Much more likely to enroll | | |
| The trial has in-person study visits that occur every four to six weeks, for at least one year. | | - Much less likely to enroll | | |
|  |  | - Less likely to enroll | | |
|  |  | - Neutral | | |
|  |  | - More likely to enroll | | |
|  |  | - Much more likely to enroll | | |
| The trial provides financial compensation for all study visits, imaging and procedures. | | - Much less likely to enroll | | |
|  |  | - Less likely to enroll | | |
|  |  | - Neutral | | |
|  |  | - More likely to enroll | | |
|  |  | - Much more likely to enroll | | |
| The trial requires participants to have a care partner, friend or family member accompany them at every study visit. | | - Much less likely to enroll | | |
|  |  | - Less likely to enroll | | |
|  |  | - Neutral | | |
|  |  | - More likely to enroll | | |
|  |  | - Much more likely to enroll | | |
| The trial provides door-to-door transportation to and from all study visits. | | - Much less likely to enroll | | |
|  |  | - Less likely to enroll | | |
|  |  | - Neutral | | |
|  |  | - More likely to enroll | | |
|  |  | - Much more likely to enroll | | |
| All of the study activities, except for the first and last one, can be done **in your home at a time and day of the week you choose**, including weekends or evenings. | | - Much less likely to enroll | | |
|  |  | - Less likely to enroll | | |
|  |  | - Neutral | | |
|  |  | - More likely to enroll | | |
|  |  | - Much more likely to enroll | | |
| The study medication is a **pill** that you will take **daily.** | | - Much less likely to enroll | | |
|  |  | - Less likely to enroll | | |
|  |  | - Neutral | | |
|  |  | - More likely to enroll | | |
|  |  | - Much more likely to enroll | | |
| The study medication is an **intravenous infusion** (medicine given in the vein). You will have to go to a hospital or clinic to get it **once a month**. | | - Much less likely to enroll | | |
|  |  | - Less likely to enroll | | |
|  |  | - Neutral | | |
|  |  | - More likely to enroll | | |
|  |  | - Much more likely to enroll | | |
| The preliminary results are in…the drug seems safe in humans. This next phase of the study is testing whether the drug is effective. | | - Much less likely to enroll | | |
|  |  | - Less likely to enroll | | |
|  |  | - Neutral | | |
|  |  | - More likely to enroll | | |
|  |  | - Much more likely to enroll | | |

Are there any things not mentioned that would make you very likely to participate in a Parkinson’s disease research study? ________________________

**Trust in Medical Researchers Scale (TIMRS)**

Please complete the survey below.

|  |  | **Strongly Agree** | **Agree** | **Neutral** | **Disagree** | **Strongly Disagree** | **I don’t know** |
| --- | --- | --- | --- | --- | --- | --- | --- |
| **1** | To get people to take part in a study, medical researchers usually do not explain all of the dangers about participation. |  |  |  |  |  |  |
| **2** | Participants should be concerned about being deceived or misled by medical researchers. |  |  |  |  |  |  |
| **3** | Usually, researchers who make mistakes try to cover them up. |  |  |  |  |  |  |
| **4** | Medical researchers act differently toward minority subjects than toward white subjects. |  |  |  |  |  |  |
| **5** | Medical researchers unfairly select minorities for their most dangerous research studies. |  |  |  |  |  |  |
| **6** | Some medical research projects are secretly designed to expose minority groups to diseases such as AIDS. |  |  |  |  |  |  |
| **7** | Medical researchers are generally honest in telling participants about different treatment options available for their conditions. |  |  |  |  |  |  |
| **8** | Usually, medical researchers tell participants everything about possible dangers. |  |  |  |  |  |  |
| **9** | All in all, medical researchers would not conduct experiments on people without their knowledge. |  |  |  |  |  |  |
| **10** | Most medical researchers would not lie to people to try to convince them to participate in a research study. |  |  |  |  |  |  |
| **11** | In general, medical researchers care more about doing their research than about the participants’ medical needs. |  |  |  |  |  |  |
| **12** | Researchers are more interested in helping their careers than in learning about health and disease. |  |  |  |  |  |  |
